# The dispensability of V_H_-V_L_ pairing and the indispensability of V_L_ domain integrity in the IgG1 secretion process

**DOI:** 10.3389/fmolb.2024.1346259

**Published:** 2024-05-02

**Authors:** Juho Choi, Yerin Jeon, Youngin Roh, Jeongyun Jang, Eunbin Lee, Luigie Villamante, Minjae Kim, Myung-Hee Kwon

**Affiliations:** ^1^ Department of Biomedical Sciences, Graduate School, Ajou University, Suwon, Republic of Korea; ^2^ Department of Microbiology, Ajou University School of Medicine, Suwon, Republic of Korea

**Keywords:** IgG, assembly, secretion, endoplasmic reticulum quality control, VH domain, VL domain

## Abstract

**Introduction:** The C_H1_ domain of IgG antibodies controls assembly and secretion, mediated by the molecular chaperone BiP via the endoplasmic reticulum protein quality control (ERQC) mechanism. However, it is not clear whether the variable domains are necessary for this process.

**Methods:** Here, we generated IgG1 antibodies in which the V domain (V_H_ and/or V_L_) was either removed or replaced, and then assessed expression, assembly, and secretion in HEK293 cells.

**Results:** All Ig variants formed a covalent linkage between the C_γ1_ and C_κ_, were successfully secreted in an assembled form. Replacement of the cognate V_κ_ with a non-secretory pseudo V_κ_ (_ψ_V_κ_) hindered secretion of individual or assembled secretion of neither heavy chains (HCs) nor light chains (LCs). The _ψ_LC (_ψ_V_κ_-C_κ_) exhibited a less folded structure compared to the wild type (wt) LC, as evidenced by enhanced stable binding to the molecular chaperone BiP and susceptibility to proteolytic degradation. Molecular dynamics simulation demonstrated dramatic alterations in overall structure of _ψ_Fab (Fd-_ψ_LC) from wt Fab.

**Discussion:** These findings suggest that V domains do not initiate HC:LC assembly and secretion; instead, the critical factor governing IgG assembly and secretion is the C_H_-C_L_ pairing. Additionally, the structural integrity of the V_L_ domain is crucial for IgG secretion. These data offer valuable insight into the design of bioactive molecules based on an IgG backbone.

## Introduction

Antibodies (Abs) comprise two heavy chains (HCs) and two light chains (LCs), the proper folding and assembly of which are prerequisite for secretion by Ab-producing cells. The endoplasmic reticulum quality control (ERQC) mechanism in these cells ensures that only correctly assembled Ig molecules are secreted, thereby preventing secretion of misfolded and misassembled immunoglobulin (Ig) proteins (Feige et al., 2010). The ERQC mechanism has been examined most extensively in the context of the IgG isotype.

Newly synthesized Ig chains assemble first as gamma H chain (γHC) dimers in the ER through interchain disulfide bonds. The C_γ1_ domain of these HC dimers remains unfolded and is retained in the ER by the molecular chaperon BiP until it binds covalently to the two folded LCs through interaction with the C_L_ domain (Bole et al., 1986; Hendershot et al., 1987). Interaction of the folded C_L_ domain with the C_γ1_ domains releases BiP and induces complete folding and oxidization of the C_γ1_ domain, thereby enabling assembly of the HC and LC into IgG (H_2_L_2_) structures prior to secretion (Lee et al., 1999; Vanhove et al., 2001; Feige et al., 2009).

The C_γ1_ domain of the HC cannot fold or form intradomain disulfide bonds in the absence of LC expression; it therefore remains a substrate for BiP. BiP-bound HCs retained in the ER eventually undergo degradation in the proteasome compartment, unless they assemble with LCs (Mains and Sibley, 1983). Thus, full-size HCs are typically not secreted without first being assembled with LCs. Deletion of C_γ1_ from IgG results in secretion of short HC dimers (V_H_-C_γ2-3_), independent of LC expression. This is likely due to bypassing of BiP-mediated ER retention (Hendershot et al., 1987; Janssens et al., 2006; Zou et al., 2007; Drabek et al., 2016; Zhang et al., 2019). HC dimers secreted without first assembling with LC, often referred to as HC-only Abs (HCAbs), can exist as C_H1_-deleted forms or as full-size HC forms. HCAbs are not commonly found in healthy mammals; one exception is the camel, which naturally expresses C_H1_-lacking HC dimers (V_H_H-C_γ2-3_) because it lacks the genes encoding the C_H1_ domain and LC (Hamers-Casterman et al., 1993). In human heavy chain disease (HCD), abnormal short HC dimers are overproduced by malignant B cells due to V_H_ gene mutations during somatic hypermutation; such mutations often cause partial or complete loss of the C_H1_ domain (Biewenga et al., 1980; Corcos et al., 2011). Certain humanized HCs bearing modified V_H_ sequences can be secreted as full-size HC dimers (Stoyle et al., 2017; Mieczkowski et al., 2020).

Conversely, most LCs can be secreted in free form without the need for HC assembly (Ambrosino et al., 1991; Dul et al., 1996; Leitzgen et al., 1997; Skowronek et al., 1998). While certain LCs require dimerization for proper folding and secretion (Leitzgen et al., 1997), many LCs can be secreted as monomers (Ambrosino et al., 1991; Dul et al., 1996; Skowronek et al., 1998). Most free LCs bind transiently to BiP through their V_L_ domain, in contrast to HCs where BiP associates stably with unfolded and reduced C_γ1_. However, LCs with specific sequences in the V_L_ form stable BiP/LC complexes, which prevents secretion of free LCs (Dul and Argon, 1990; Ma et al., 1990; Knittler et al., 1995; Skowronek et al., 1998).

Studies involving point mutations demonstrate that the V_H_ and/or V_L_ domain sequences have a marked impact on the efficiency of Ig assembly and secretion (Wu et al., 1983; Dul and Argon, 1990; Dul et al., 1992; Rocca et al., 1993; Chen et al., 1994; Horwitz et al., 1994; Martin et al., 1996; Wiens et al., 2001; Whitcomb et al., 2003; Stoyle et al., 2017; Mieczkowski et al., 2020); however, the pivotal role of V_H_ and V_L_ domains during the IgG assembly process remains uncertain, particularly regarding whether the pairing of V_H_/V_L_ provides a signal that initiates covalent assembly of HC and LC.

The aim of the present study was to investigate the intrinsic significance and role of the V_H_ and/or V_L_ domains during the process of IgG secretion and assembly by deleting V domain (s), rather than focusing on mutational effects. Plasmid vectors encoding various Ig fragments were designed based on the IgG1/κ molecule, some lacking V_H_ or V_κ_, or both, while others contained pseudo V_κ_ (_ψ_V_κ_). We then assessed expression and secretion of these Ig fragments by HEK293 cells. We also investigated the BiP binding and resistance to proteolytic degradation of _ψ_V_κ_-harboring LC (_ψ_LC), and the influence of _ψ_V_κ_ domain on the overall structure of Fab format by molecular dynamics simulation. The results show that the V_H_ or V_L_ domain, or pairing of V_H_/V_L_, are not necessary for the IgG assembly and secretion process. Instead, the decisive factor governing this process is the pairing between C_H_ and C_L_, while the structural integrity of the V_L_ domain is crucial.

## Materials and methods

### Plasmid construction

Plasmid vectors expressing 6C407 IgG1 and its variants were generated from plasmids KV10 and KV12, which differ only in terms of the restriction enzyme sites flanking the HC genes (Kim et al., 2019; Seo et al., 2020). 6C407 IgG1, a chimeric Ab bearing V regions (V_H_ and V_κ_) of mouse origin and C regions (C_γ_ and C_κ_) of human origin, is specific for the KIFC1 antigen (Seo et al., 2020). In the KV10 plasmid, the V_H_ and C_H_ chain genes are flanked by *Mlu*I/*Nhe*I and *Nhe*I/*BamH*I restriction enzyme sites, respectively, while the V_L_ and C_L_ genes were flanked by *Bgl*II/*BsiW*I and *BsiW*I/*EcoR*I, respectively. In KV12, the V_H_ and C_H_ chains are flanked by *Mlu*I/*Nhe*I and *Nhe*I/*Hind*III restriction enzyme sites, respectively. These plasmids act as a cassette vector that permits cloning of the HC and LC genes harboring upstream leader sequences into specific cloning sites, followed by simultaneous expression of HC and LC under the control of two individual CMV promoters (P_CMV_). By replacing or deleting specific domain genes from the pre-existing KV10-6C407 IgG1 (Seo et al., 2020), a diverse set of plasmids, including KV10-[HC + C_κ_], KV10-[C_γ1-3_ + LC], KV10-[HC + _ψ_LC], KV10-[C_γ1-3_ + _ψ_LC], KV10-[C_γ1-3_ + LC], KV12-[HC], KV10-[C_γ1-3_], and KV10-[Fc]. KV10-[C_γ1-3_ + C_κ_], also known as KV10-IgCw-γκ, was generated (Kim et al., 2019). The _ψ_V_κ_ sequence (here designated as 2C281 _ψ_V_κ_) is available from GenBank (accession no. MH638370.1).

### Cell culture

Human embryonic kidney 293 (HEK293) cells (ATCC; cat# CRL-1573) were cultured in Dulbecco’s Modified Eagle’s Medium (DMEM, Welgene; cat# LM 001-05) supplemented with 10% fetal bovine serum, 100 U/mL penicillin, and 100 μg/mL streptomycin in a 5% CO_2_ humidified incubator at 37°C.

### Transfection of plasmids encoding Ig fragments

Cells were seeded in 60 mm dishes at a density of 1 × 10^6^ cells/dish 24 h prior to transfection with plasmids. Plasmid DNA (4 μg) was pre-incubated at room temperature for 10 min with polyethylenimine (PEI) reagent (12 μg), and then added to the seeded cells in 200 μL of Opti-MEM (ThermoFisher Scientific; cat# 31985-070). Dishes were incubated at 37°C for 48 h.

### Preparation of supernatant and cell lysates

At 48 h post-transfection, the cell culture was collected and a clear supernatant obtained by centrifugation. Transfected cells were washed three times with cold PBS and then harvested in cold PBS using a scraper. The collected cells were centrifuged at 850 *g* at 4°C for 5 min, and then lysed with IP Lysis Buffer (0.025 M Tris, 0.15 M NaCl, 0.001 M EDTA, 1% NP-40, 5% glycerol, pH 7.4) containing a protease inhibitor cocktail (Roche; cat# 11697498001). Supernatants were obtained by centrifugation at 16,000 × g at 4°C for 10 min. The protein concentration in the cell lysates was measured using a BCA Protein assay kit (Thermo Fisher Scientific; cat# 23227).

### Immunoprecipitation (IP)

Aliquots of cell lysate (250 μg) prepared as described above were subjected to immunoprecipitation at 4°C for 18 h using anti-HA agarose (Thermo Fisher Scientific; cat# 26181) and KappaXP-Agarose (Thermo Fisher Scientific; cat# 2943212005). After washing the resin, immunoprecipitated proteins were eluted by heating at 100°C for 10 min, separated by SDS-PAGE, and then analyzed by immunoblotting.

### SDS-PAGE and immunoblotting

SDS-PAGE was performed on 4%–20% gradient gels under reducing and non-reducing conditions, and resolved proteins were transferred to polyvinylidene fluoride (PVDF) membranes. Goat anti-human IgG-Fc (abcam; cat# ab97221) and goat anti-human C_κ_ (Thermo Fisher Scientific; cat# 31129) were used to detect human IgG-Fc and κ LC, respectively. A rabbit anti-goat IgG-HRP Ab was used to detect the bound primary Abs. GAPDH was detected by probing the membrane with primary mouse anti-GAPDH (Santa Cruz Biotechnology; cat# sc-322330) Ab, followed by horse anti-mouse IgG-horseradish peroxidase (Cell Signaling; cat# 7076). Immunoreactive proteins were visualized using an ECL Kit (GE Healthcare, Cat. No. RPN2106).

### Enzyme-linked immunosorbent assay (ELISA)

An indirect sandwich enzyme-linked immunosorbent assay (ELISA) was performed to assess the association between HC and LC in cell lysates. Briefly, 96-well polystyrene plates (Thermo Fisher Scientific; cat# 439454) were coated overnight at 4°C with 100 μL (2 μg/mL) of goat anti-human IgG-Fc Ab (abcam; cat# ab97221) as the capture Ab. After three washes with TBST, the wells were blocked for 1 h at room temperature with 3% BSA. Next, the wells were incubated for 1 h at room temperature with lysates of transfected cells (40 μg/well), followed by rabbit anti-human IgG κ light chain Ab (abcam; cat# ab125919) as the detection Ab, and an alkaline phosphatase-conjugated goat anti-rabbit IgG Ab (Thermo Fisher Scientific; cat# 31341). After each incubation step, the wells were washed three times with TBST (20 mM Tris-HCl, 150 mM NaCl, 0.05% Tween 20, pH 7.4). Color development was achieved by adding p-nitrophenyl phosphate substrate solution (1 mg/mL prepared in 0.1 M glycine, 1 mM ZnCl_2_, and 1 mM MgCl_2_, pH 10.3) to each well. Absorbance at 405 nm was measured in an Epoch2 microplate reader (BioTek).

### Establishment of HEK293 cells expressing HA-tagged BiP

DNA encoding HA-tagged BiP was cloned into the lentiviral vector pCDH-CMV-MCS-EF1-Puro (System Biosciences; cat# CD510B-1). Lentivirus particles were obtained by co-transfecting HEK293T packaging cells (2 × 10^6^) with pCDH-CMV-BiP-HA-puro (4 μg), GAG-pol (3 μg), and pVSV-G (1 μg) DNA, which was premixed in a tube containing PEI reagent (16 μg). Culture medium containing recombinant lentivirus was harvested at 48 h post-transfection. Then, 1 mL of culture medium containing recombinant lentivirus was used to infect HEK293 cells seeded overnight into 60 mm dishes (2 × 10^6^ cells/dish) containing 5 mL of fresh culture medium supplemented with 10 μg/mL polybrene. At 48 h post-infection, infected cells were selected by replacing the medium with DMEM supplemented with 5 μg/mL puromycin.

### Protease susceptibility assay

Lysates from HEK293 cells transfected with LCs were prepared as described above in the ‘*Preparation of supernatant and cell lysates*’ section. Aliquots (500 μg) of lysate underwent IP at 4°C for 18 h using KappaXP-Agarose. After washing the resin, IP proteins were eluted with 0.1 M glycine (pH 3.0) and the pH of eluted proteins was adjusted to 7 with 1 M Tris (pH 9.5). Neutralized eluates (20 μL) were treated with trypsin (5 mg/mL) to achieve a final concentration of 0.2 mg/mL. Trypsin digestion was performed at 37°C for 0.5–10 min and quenched immediately on ice after each reaction. Samples were resolved by SDS-PAGE, followed by immunoblotting with an anti-C_κ_ antibody. Band intensity was quantified using ImageJ software and normalized to the undigested protein.

### Computer-assisted structure prediction of Fabs

Protein structure modeling of Fab fragments (wild-type (wt) Fab and _ψ_Fab) was performed using Ab modeler embedded in Biovia Discovery Studio 2020 (Modeler ver. 9.22). The amino acid sequences of two HCs and LCs were annotated by the ImMunoGeneTics information system (IMGT) numbering scheme. Crystal structures M2177 (PDB ID: 5TL5) and 13A9 (PDB ID: 6DDR) were used as templates for the overall structures of the wt Fab and _ψ_Fab, respectively. The LC structures of Abs 14.1 (PDB ID: 5FB8) and 13A9 (PDB ID: 6DDR) were used as templates for the LC portions of wt and _ψ_Fab, while the HC structure of Ab 48G7 (PDB ID: 1AJ7) served as the template for generating HC structures for wt Fab and _ψ_Fab. Template structures were selected based on sequence similarity. The “Identify Framework Templates,” “Model Ab Framework,” and “Model Ab Loop” protocols in Discovery Studio were applied sequentially to generate structures for both Fabs. The generated models were ranked according to their probability density function (PDF) total energy.

The model with the lowest total potential energy determined by PDF analysis was subjected to explicit-water molecular dynamics (MD) simulation using the Discovery Studio 2020 software, which incorporates the CHARMm force field. Solvation and charge neutralization of the Fabs were followed by energy minimization by the built-in Smart Minimizer module within the Discovery Studio. The energy minimized protein–solvent system was gradually heated from 0 to 300 K at 1 atm pressure over a period of 100 ps. Then equilibration simulation of the energy minimized protein–solvent system run at 300 K for 200 ps. Finally, production simulation of the equilibrated protein–solvent system was conducted, running for 1 ns in the NVT (constant number of particles, volume, and temperature) ensemble. During this phase, structural coordinates of the system were saved at intervals of 2 ps. The accuracy of the predicted Fab models was validated using Ramachandran plots.

From the predicted Fab structure, we calculated the Fab elbow angle and visually presented it using the PyMOL software (Schrödinger LLC. 2021. the PyMOL molecular graphics system, version 2.5.0, https://www.pymolwiki.org/index.php/Elbow_angle), which allows for the calculation of the Fab elbow angle and simultaneous visualization of the two pseudo-dyad axes on the Fab structure.

## Results

### V_H_ and V_L_ pairing is unnecessary for assembly and secretion of IgG

**FIGURE 1:**
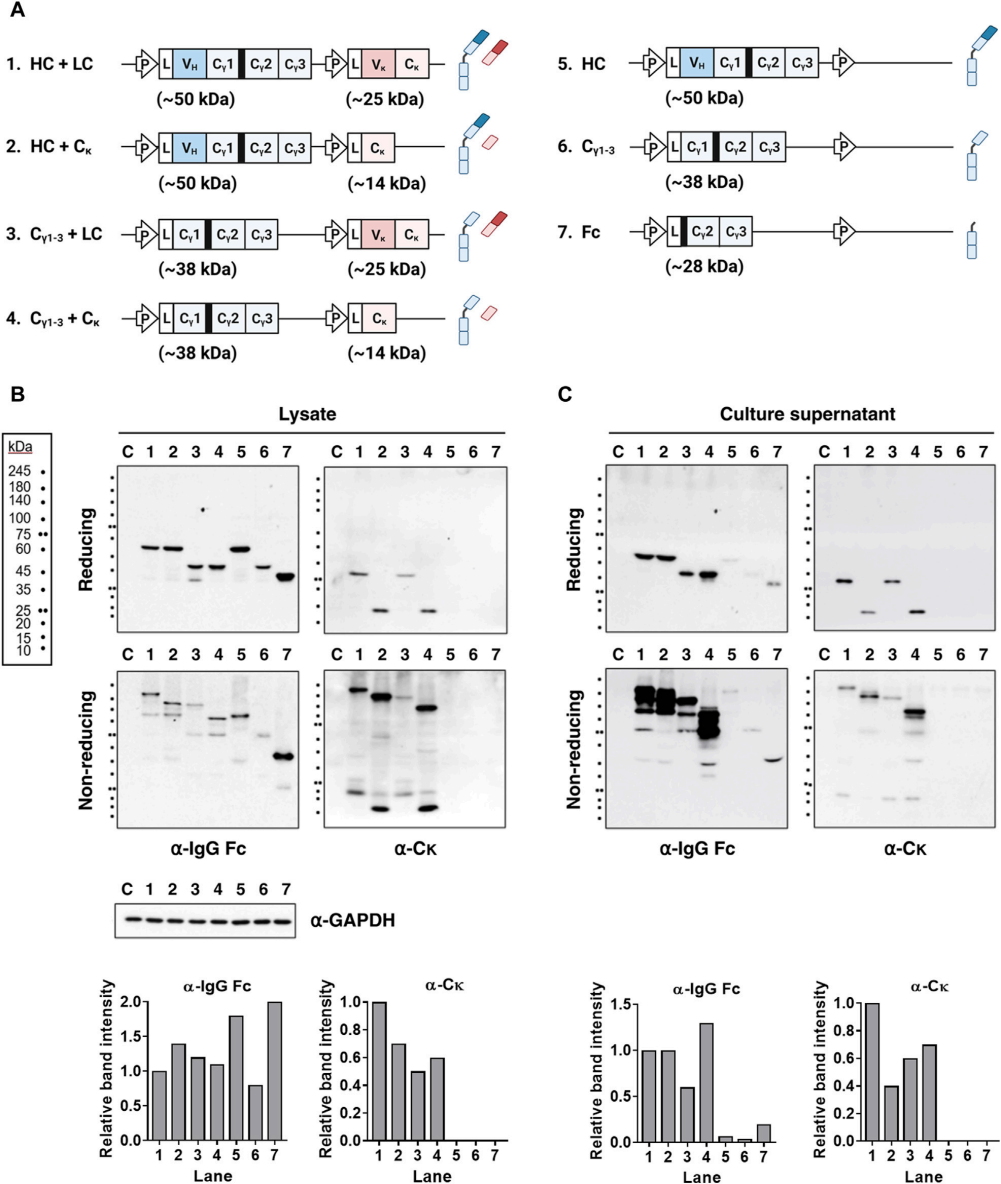
Covalent association between the C_γ1_ and C_κ_ domains is sufficient for secretion of fully assembled Igs, regardless of the presence of the V domains. **(A)** Schematic representation of the plasmid vectors and Ig chain (or domain) proteins encoded by the corresponding Ig genes. The expected molecular weights of the individual subunits are indicated. The hinge region of human γ heavy chain is shown as a thick black bar upstream of the C_γ2_ region. L, leader sequence; P, cytomegalovirus promoter. **(B,C)** Immunoblot analysis of Ig subunits. The lysates **(B)** and supernatant **(C)** of HEK293 transfectants were resolved in reducing and non-reducing SDS-PAGE gels. The resolved Ig proteins were probed with antibodies specific for human IgG-Fc and C_κ_ region. The bar graph below each gel image displays the relative intensity of bands detected by the specified antibodies under reducing conditions. Lanes 1–7 contain cell lysates obtained after lysis if cells transfected with each plasmids labeled as in **(A)**. Lane C represents the non-transfected control. GAPDH was used as a loading control for protein normalization.

First, we investigated whether the presence of the V_H_ and/or V_L_ domains of IgG affects secretion of fully assembled Ig. We did this by generating domain-deleted variants using a single-vector strategy to co-express HC and LCs (Figure 1A). Subsequently, lysates and supernatants of transfected cells were analyzed under both reducing and non-reducing denaturing conditions. The results revealed that compared with wt IgG HC, expression levels of HCs and LCs were similar in the absence of the V_H_, V_L_, or both domains (Figure 1B, lanes 1–4). Analysis of the supernatants revealed that Igs lacking the V_H_, V_L_, or both domains were secreted in their fully assembled form (Figure 1C, lanes 1–4). This indicates that the V_H_ and V_L_ chains, or their paired form (V_H_/V_L_), are dispensable for the secretory competence of IgG. Rather, the C_γ1_ and C_κ_ domains are the determining factor.

Previously, it was thought that full-size HC proteins could not be secreted independently of the LC. However, we found that they were secreted at very low, nearly undetectable levels, rather than not at all. The secreted HC-only protein appeared as a multimer (>180 kDa), in contrast to the monomers and HC dimers detected in cell lysates (Figures 1B, C, lane 5). In the presence of the C_κ_ chain, the C_γ1-3_ chains were secreted in a covalently assembled form (as 2C_γ1-3_2C_κ_ tetramers), at a level almost equivalent to that of IgG (based on protein band intensity); however, the C_γ1-3_ chains expressed in the absence of LC were secreted as dimers (∼86 kDa), but at a very low level (Figure 1C, lanes 4 and 6). This aligns with our earlier discovery that co-expression of C_γ1-3_ and C_κ_ chains in HEK293F cells produces fully assembled IgG-like structures, at concentrations 30 times greater than that of C_γ1-3_ alone (Kim et al., 2019). The C_γ2-3_ chain (an Fc fragment containing the hinge region) was secreted as a dimer (∼56 kDa) at a significantly higher level than the C_γ1-3_ chain (Figure 1C, lane 7). The difference in secretion of C_γ2-3_ and C_γ1-3_ is likely due to the ability of the C_γ2-3_ chain to bypass C_γ1_-mediated ERQC, which involves C_κ_ domains.

### An aberrant _ψ_V_κ_ domain disrupts secretion of Ig

**FIGURE 2:**
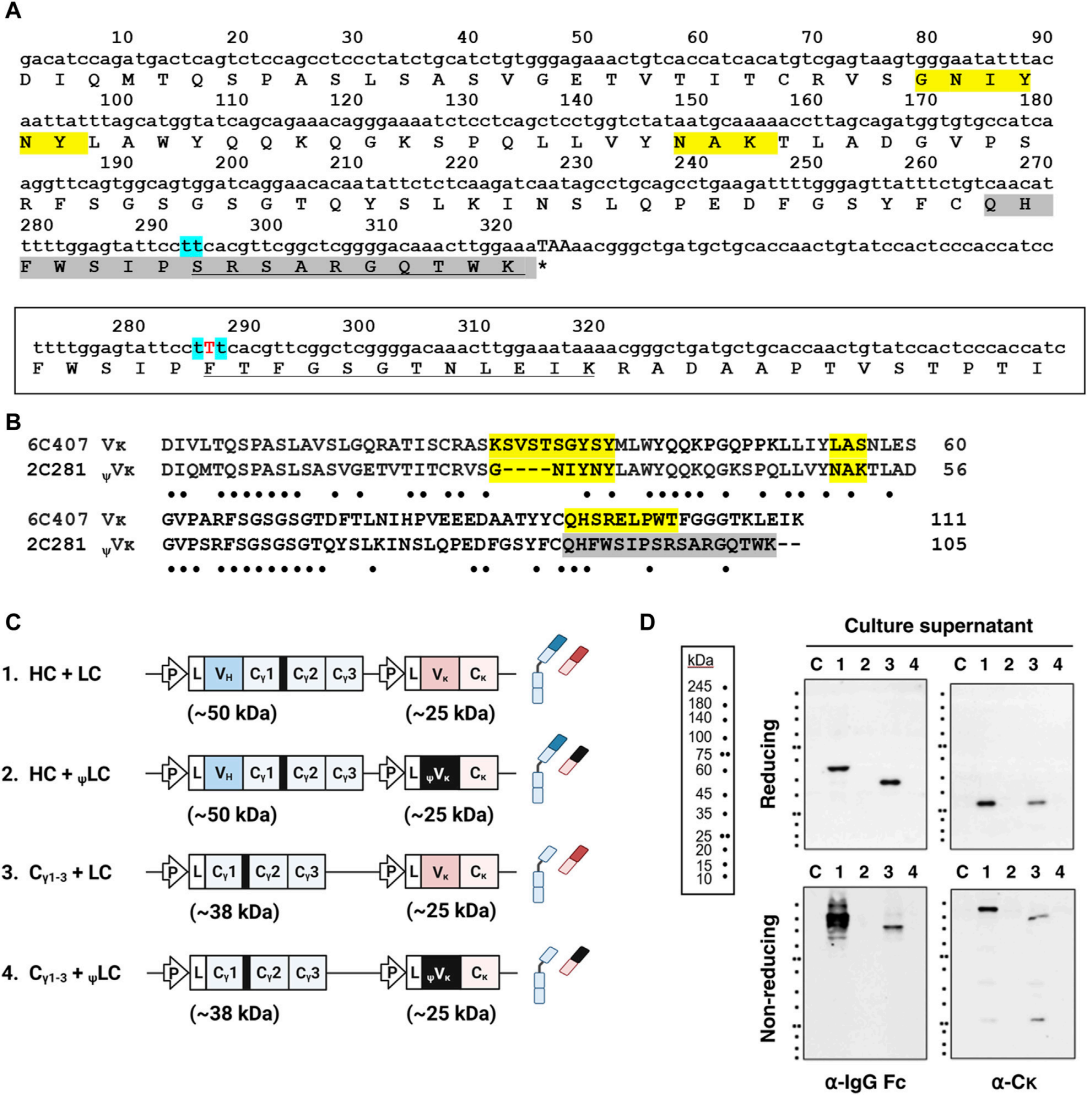
Replacement of V_κ_ with the _ψ_V_κ_ domain disrupts secretion of Ig molecules. **(A)** Nucleotide and amino acid sequences of 2C281 _ψ_V_κ_. CDR residues according to the IMGT (International ImMunoGeneTics) information system shaded yellow. Due to a frame shift mutation in CDR3, the IMGT system does not define CDR3 of 2C281 _ψ_V_κ_. Consequently, regions beyond FR3 are shaded in grey. Mint-colored shading marks the site with presumed base deletion (s). Amino acid sequence affected by base (s) deletion-induced reading-frame shift is underlined. Asterisk indicates stop codon. **(B)** V_κ_ sequence alignment. Sequences of 2C281 _ψ_V_κ_ and true V_κ_ regions from 6C407 clone aligned using the Clustal Omega server (https://www.ebi.ac.uk/Tools/msa/clustalo/). Absent residues denoted by dash. Dots indicate the positions of conserved residues between the sequences. **(C)** Schematic representation of the plasmid vectors and genes encoding the corresponding Ig chain (or domain) proteins. The hinge region of the human γ1 heavy chain is always present upstream of the C_γ2_ region, and is shown here as a thick black bar. L, leader sequence; P, cytomegalovirus promoter. **(D)** Immunoblot analysis of culture supernatants. Lanes 1–4 contain supernatants obtained from cells transfected with each plasmid labeled as in **(C)**. Lane C represents the non-transfected control.

While our observations indicate that the V_H_/V_L_ pairing is not critical for assembly and secretion of IgG (Figure 1), we sought to further investigate the impact of improper V_κ_ domain on IgG secretion, spurred by reports of V_H_-induced alterations in C_γ1_ structure that led to HC-only antibody secretion through prevention of BiP binding (Stoyle et al., 2017; Mieczkowski et al., 2020). We hypothesized a similar scenario where distinct V_κ_ variants could induce changes in C_κ_ structure and potentially impact IgG secretion. To investigate this, we substituted the native V_κ_ domain with a pseudo type V_κ_ (_ψ_V_κ_) derived from 2C281 hybridoma clone, which is characterized by the absence of defined FR4 segment resulting from a frame shift mutation in CDR3 (Figures 2A, B). Given FR4 is positioned just upstream of the C_κ_ domain, it is presumed to exert significant influence on C_κ_ structure. We designated LC harboring _ψ_V_κ_ as _ψ_LC. We then investigated their secretion into the supernatant of four HEK293 cell transfectants: [HC + LC], [C_γ1-3_ chain + LC], [HC + _ψ_LC], and [C_γ1-3_ chain + _ψ_LC] (Figures 2C, D).

When HCs and _ψ_LCs were co-expressed, neither were secreted into the culture supernatant, whether individually, as assembly intermediates, or in their fully assembled form (Figure 2D, lane 2). Surprisingly, the same outcome was observed when co-expressing the C_γ1-3_ chain with _ψ_LCs (Figure 2D, lane 4). The reason why the C_γ1-3_ chain did not get secreted when co-expressed with _ψ_LC, in contrast to its successful expression in assembled form with wt LC (as shown in Figure 1), is likely because the _ψ_V_κ_ domain induces unfavorable changes in the downstream C_κ_ structure. The altered C_κ_ domain may fail to trigger BiP dissociation from C_γ1_ and proper C_γ1_ folding, leading to ultimate failure of secretion as an assembled Ig.

### An aberrant V_κ_ domain prevents the LC from associating with the HC

**FIGURE 3:**
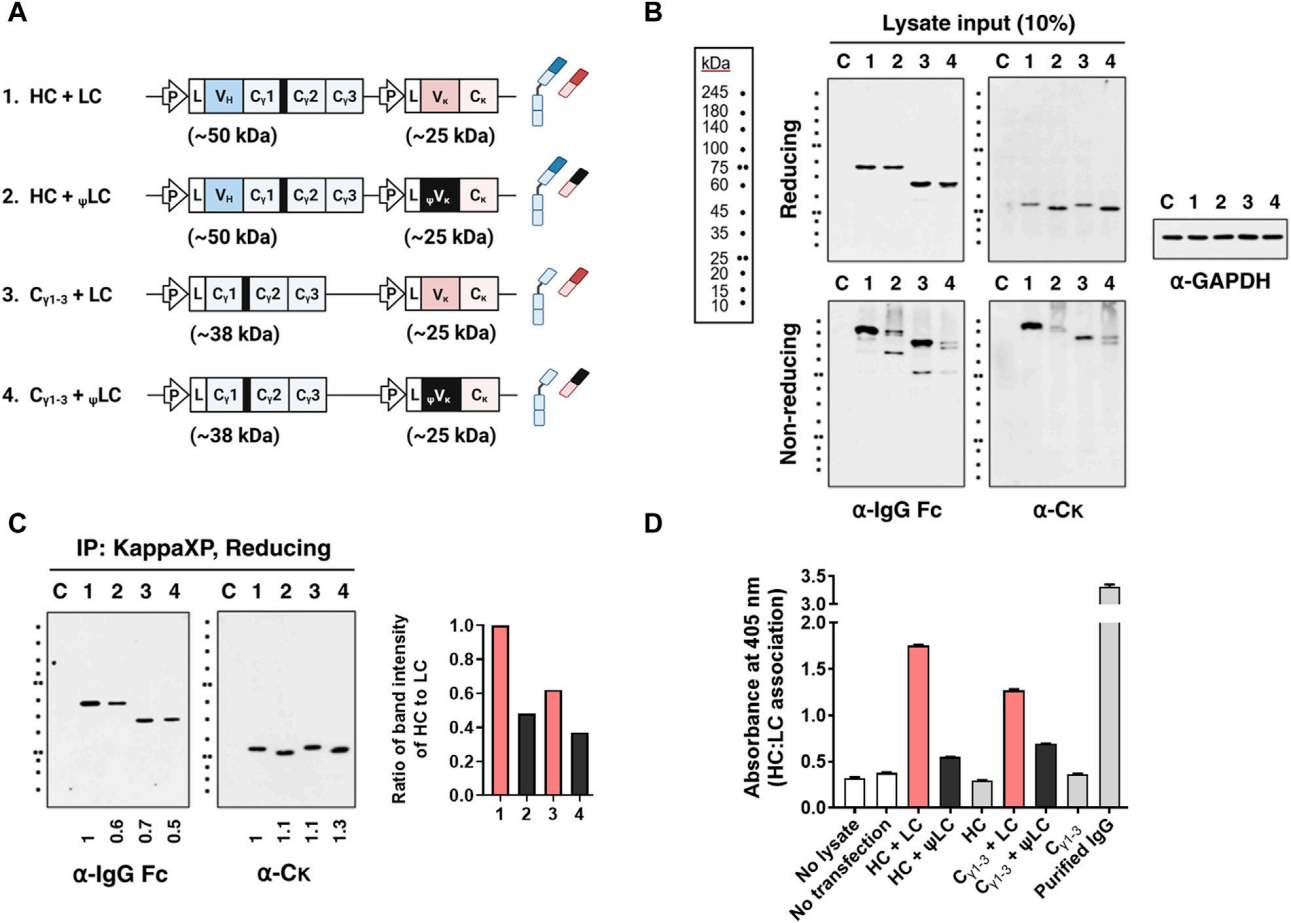
The presence of an improper V_κ_ domain hinders assembly of HC and LC. **(A)** Schematic representation of the plasmid vectors and genes encoding the corresponding Ig chain (or domain) proteins. The hinge region of human γ1 heavy chain is always present upstream of the C_γ2_ region, and is shown here as a thick black bar. L, leader sequence; P, cytomegalovirus promoter. **(B,C)** Immunoblot and immunoprecipitation (IP) analyses of Ig subunits in cell lysates. Lysates of HEK293 transfectants were immunoprecipitated with KappaXP-Agarose, which captures the C_κ_ domain. Input samples and IP samples were separated by reducing and/or non-reducing SDS-PAGE, and subsequently analyzed by immunoblotting. **(B)** The input represents 10% of the total amount of lysate used for IP. GAPDH was used as an internal loading control. **(C)** Co-IP analysis of HC and LC in lysates from HEK293 transfectants. Numbers below the Western blot images represent protein band intensity, which was analyzed with ImageJ software and normalized to lane 1. The bar graph shows the ratio of the HC band intensity to that of the pulled-down LC. **(D)** Evaluation of H:L chain association by sandwich ELISA. Lysates of transfectants were placed in wells coated with goat anti-human IgG/Fc, and bound LCs were detected with rabbit anti-human C_κ_ followed by an AP-conjugated anti-rabbit IgG Ab. The data are expressed as the mean ± standard deviation of triplicate samples, and are representative of a single experiment from a series of three independent experiments.

To determine whether the expressed aberrant _ψ_LC can associate with HC, we assessed expression as well as assembly of HC-LC in lysates of four HEK293 cell transfectants: [HC + LC], [C_γ1-3_ chain + LC], [HC + _ψ_LC], and [C_γ1-3_ chain + _ψ_LC] (Figure 3A). When HCs and _ψ_LCs were co-expressed, both chains were expressed within the cells to a degree similar to that of IgG, as seen in the input analysis under reducing conditions (Figure 3B). However, efficiency of correct and covalent HC-_ψ_LC assembly within the cells was lower than that of wt IgG, as observed in the input analysis under non-reducing conditions. This was evident from a decrease in the size of IgG band (150 kDa), and the appearance of multiple smeared protein bands that were larger than the molecular weight of IgG, in the input analysis under non-reducing conditions (Figure 3B, lower panels, lane 2). The same observation was made when the C_γ1-3_ chain, rather than full-size HC, was co-expressed with _ψ_LCs (Figure 3B, lower panels, lane 4). The reduced efficiency of HC-_ψ_LC assembly was confirmed by IP-immunoblotting and ELISA. The quantity of HC (as well as C_γ1-3_ chain) pulled down by LC C_κ_ exceeded that pulled down by _ψ_LC C_κ_. Protein band intensity was normalized to IgG HC (Figure 3C, lane 1), and presented as a graph alongside the gel image. The same lysates used in Figure 3B were utilized for the ELISA. For the ELISA, the wells were coated with an anti-Fc Ab for capture, followed by incubation of cell lysates with an anti-C_κ_ Ab for detection. We noted a 52% reduction in HC and _ψ_LC assembly compared with full-size HC and LC. In addition, assembly of the C_γ1-3_ chain and _ψ_LC decreased by 40% in comparison to C_γ1-3_ chain and the LC (Figure 3D). Taken together, the data suggest that _ψ_LC associates less with the HC or C_γ1-3_ chain, despite both being expressed within cells.

### The _ψ_LCs exhibit stable interactions with BiP molecules and higher sensitivity to protease compared to wt LCs

**FIGURE 4:**
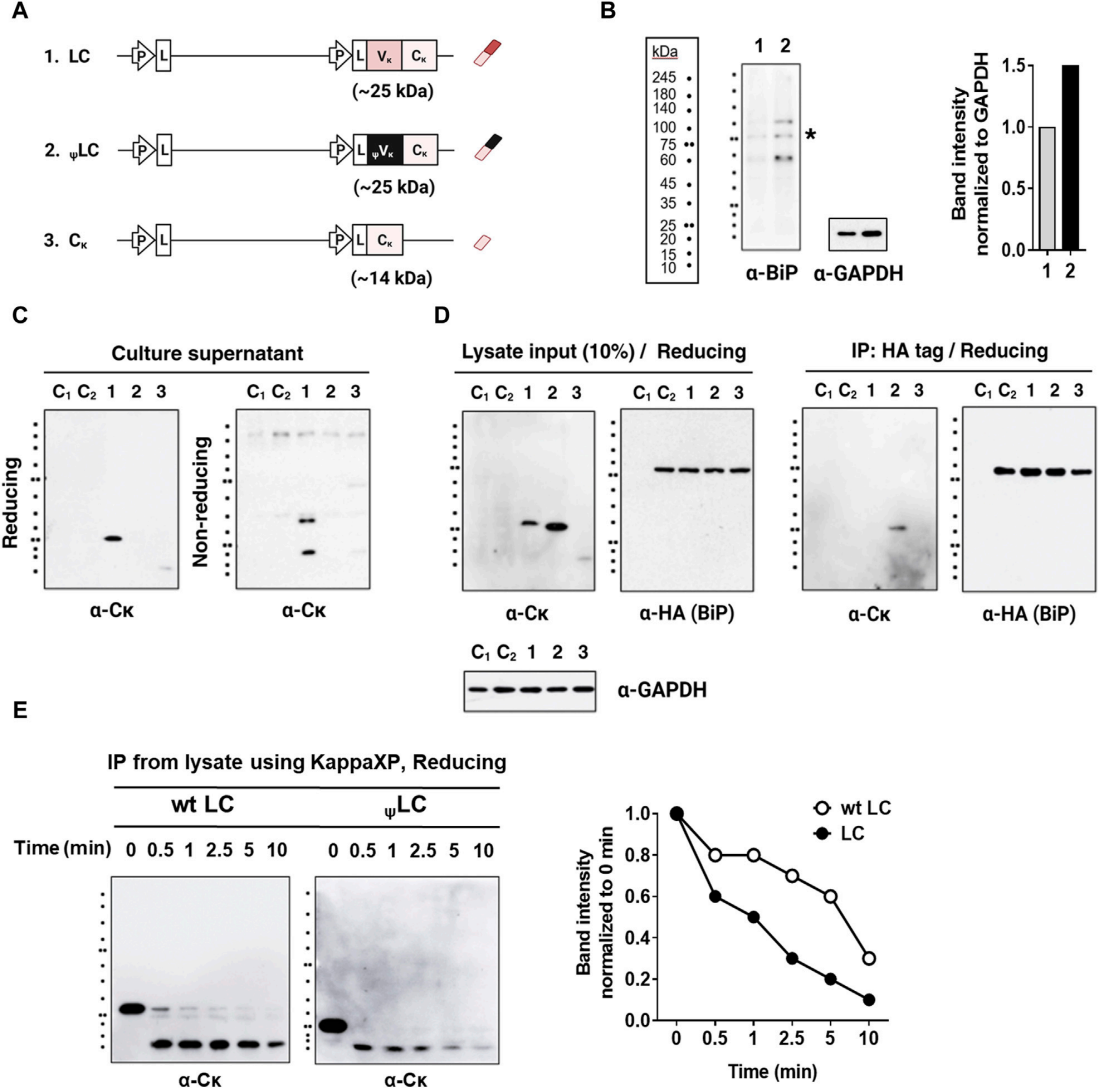
Interaction of LCs with BiP in HEK293 cells stably expressing HA-tagged BiP (HEK293-BiP). **(A)** Schematic representation of the plasmid vectors and genes encoding the corresponding Ig chain (or domain) proteins. **(B)** BiP detected using anti-BiP antibody in wild-type HEK293 cells (lane 1) and HEK293 cells transduced with the BiP-HA lentiviral particles (lane 2). The asterisk indicates the band corresponding to the size of BiP **(C)** Immunoblot analysis of LCs in the culture supernatant of HEK293-BiP. Forty-eight post-transfection culture supernatants from HEK293 transfectants were resolved in reducing and non-reducing SDS-PAGE gels. **(D)** Co-IP of LC and BiP in lysates from HEK293-BiP cells transfected with LCs. Co-immunoprecipitation was performed using an anti-HA agarose. Proteins in the extract (input = 10% of the lysate) and pull-down fractions (IP) were resolved by reducing SDS-PAGE and probed with Abs specific for human IgG-Fc, the C_κ_ region, and the HA tag. Lane C_1_, non-transfected HEK293 control; Lane C_2_, non-transfected HEK293-BiP control. **(E)** Protease susceptibility assay. wt LCs and _ψ_LCs were enriched by IP of lysates from the respective HEK293 transfectants using KappaXP-Agarose. The eluates were treated with protease trypsin for the indicated times and resolved by SDS-PAGE, followed by immunoblotting with an anti-C_κ_ antibody. The intensity of bands from wt LC and _ψ_LC proteins treated with trypsin was normalized to the untreated lane (0 min) and displayed as a graph alongside the gel image. The graph was generated from the cumulative intensity sum of three protein bands in each lane.

Most LCs can be secreted on their own, independently of their association with HCs; these are designated as secretory-competent LCs. They then associate transiently with BiP through their V_L_ domain, and can be secreted as monomers or homodimers. However, certain secretory-incompetent LCs bind irreversibly to BiP and cannot be secreted due to factors such as aggregation, incomplete folding, the presence of free thiol groups, and an inability to form homodimers; all of these characteristics are determined by the V_L_ amino acid sequence (Reddy et al., 1996; Leitzgen et al., 1997; Skowronek et al., 1998). Therefore, we asked whether diminished association efficiency between _ψ_LC and HC stemmed from the inability of the _ψ_LC protein to achieve complete folding, causing BiP to retain _ψ_LC within the cells. To investigate this, we conducted a study examining secretion, expression, and BiP binding within HEK293 cells. This cell line stably expressed BiP fused to an HA tag, and was transfected with genes encoding wt LC, _ψ_LC, and C_κ_ (as depicted in Figure 4A). The HEK293 cells transduced with the BiP-HA lentiviral particles exhibited a 1.5-fold increase in BiP expression (both endogenous BiP and BiP-HA) compared to wild-type HEK293 cells (containing only endogenous BiP) (Figure 4B).

Notably, _ψ_LCs were not secreted into the culture supernatant, in stark contrast to wt LC (Figure 4C). This observation, in conjunction with previous findings (shown in Figure 2), suggests that _ψ_LC is inherently secretion-incompetent, regardless of the presence or absence of HC. We also observed that wt LC was secreted in both monomeric and dimeric forms, while C_κ_ was secreted as a dimer (Figure 4C, lanes 1). It is intriguing that wt LC is secreted as a mixture of these two forms, contrary to previous reports that some LCs are secreted as dimers (Leitzgen et al., 1997) while others are monomers (Ambrosino et al., 1991; Dul et al., 1996; Skowronek et al., 1998). Secretion of single C_κ_ domains was observed at a level barely detectable in the absence of HC expression, unlike wt LC (V_κ_-C_κ_). Regarding cellular expression, _ψ_LCs exhibited higher expression than wt LC, as observed in the input analysis (Figure 4D, left panel). BiP binding to all three chains was confirmed through immunoprecipitation. Only _ψ_LC was pulled down by the HA tag, indicating that _ψ_LCs are stably bound to BiP within cells (Figure 4D, right panel). Our findings align with those of a previous report indicating that V_L_ domains dictate the physical stability of BiP/LC complexes (Skowronek et al., 1998). Indeed, several mutant LCs bind BiP more avidly than their wt counterparts (Ma et al., 1990; Knittler et al., 1995). Stable binding of BiP to _ψ_LC suggests that the _ψ_LC structure possesses an incomplete conformation that cannot be secreted. In our experimental setup, we could not ascertain whether BiP binds exclusively to the _ψ_V_κ_ domain of _ψ_LC, or if it also interacts with the C_κ_ domain, which might undergo structural changes via the V_κ_/C_κ_ interface.

We conducted a protease susceptibility assay to compare the folding states of wt LC and _ψ_LC in cells. Lysates from HEK293 transfectants were immunoprecipitated with KappaXP-Agarose. The eluates treated with the broad specificity protease trypsin. Subsequently, immunoblotting was conducted using an anti-C_κ_ antibody for detection (Figure 4E). _ψ_LCs were degraded more rapidly than wt LC, indicating its more unstructured state compared to wt LC.

### _ψ_V_κ_ induces structural changes in the Fab

**FIGURE 5:**
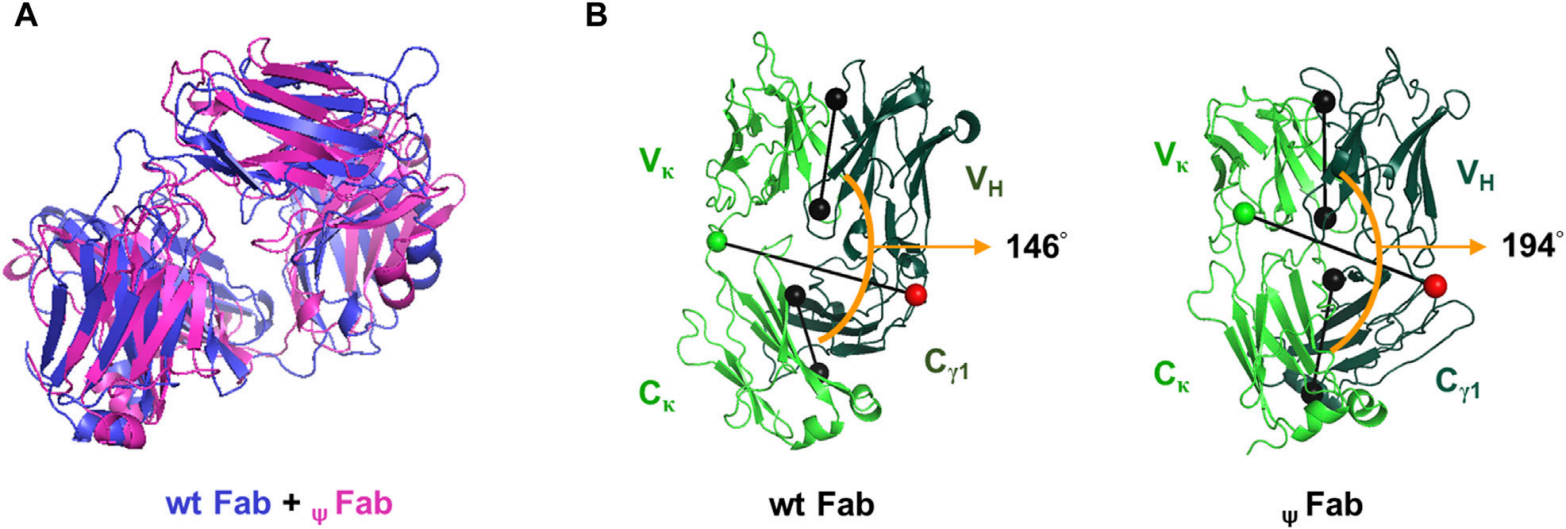
Ribbon diagrams illustrating the predicted Fab structures. **(A)** Superposition of wt Fab and _ψ_Fab structures, modeled using Discovery Studio 2020 (Modeler ver. 9.22). The Fab molecules are represented by blue (wt Fab) and pink (_ψ_Fab) ribbons. **(B)** The Fab elbow angle was calculated and represented visually by PyMOL software (version 2.5.0). Black dumbbells pass through the center of mass of the V and C domains of each Fab. The orange arc denotes the Fab elbow angles. Green and red dumbbells represent residues used to separate the V and C domains, with a green ball representing the LC and a red ball representing the HC.

To better understand why neither _ψ_LC was secreted, either individually or in assembled form, despite a partial covalent association between HC and _ψ_LC in cell lysates (Figures 3, 4), we used a computer-assisted approach to explore the structural impact of replacing the cognate V_κ_ with _ψ_V_κ_ in the Fab format, designating it as _ψ_Fab. Discovery Studio 2020 software was used to generate three-dimensional structures of both the wt Fab and _ψ_Fab proteins; it then analyzed their structural dissimilarity by measuring the root mean square deviation (RMSD) and Fab elbow angle (Figure 5). The RMSD is a measure of the average distance between the backbone C-alpha atoms of superimposed molecules. Two identical structures yield an RMSD value of zero (a distant unit), while differing structures present values proportionate to their dissimilarity. The RMSD based on superimposition of wt Fab and _ψ_Fab structures was calculated as 18.3115 Å. The Fab elbow angle is the angle between the two pseudo-dyad axes relating the variable domains (V_H_ and V_κ_) and the constant domains (C_γ1_ and C_κ_) It is a valuable indicator of the overall topology of the Fab fragment. The distributions of elbow angles vary distinctly depending on the type of Fab light chain. Kappa Fab typically ranges from 125° to 195°, with a prevalence of 135°–145°, while lambda Fab ranges from 115° to 225°, favoring 185°–195° (Stanfield et al., 2006). In this study, the kappa Fab elbow angle measured 146° for wt Fab and 194° for _ψ_Fab, showing notable distinction in the relative positioning of V and C domains. Essentially, this reflects a difference in overall arrangement of the HC and LC domains of the Fab.

Taken together, we suggest that the _ψ_V_κ_ domain might distort the downstream C_κ_ structure, making the altered C_κ_ domain lose the ability to release BiP from the C_γ1_ domain, resulting in failure of complete C_H_ folding and secretion as an assembled IgG. The modified C_κ_ domain may also lead to loss of LC secretion and hinder covalent binding with C_γ1_. We ruled out the possibility that the _ψ_V_κ_ domain has a detrimental effect on the V_H_ structure at the interface, which would then impact downstream C_γ1_, maintaining BiP binding and preventing secretion of assembled Ig. This is supported by the fact that co-expression of the _ψ_LC and C_γ1-3_ chains did not result in the secretion of assembled Ig (Figure 2), and _ψ_LC exhibited poor association with C_γ1-3_ chains (Figure 3). Thus, it seems that BiP-bound _ψ_LCs might have disrupted the entire IgG secretion process.

## Discussion

While covalent assembly between C_H1_ and C_L_, as well as the functions of BiP-mediated ERQC mechanisms during the IgG assembly and secretion process, have been studied comprehensively at the molecular level, our understanding of the role of the V domains in this process remains insufficient. It is suggested that assembly of HC and LC is initiated by non-covalent interactions between the V_H_ and V_L_ domains, which may trigger dissociation from BiP, thereby allowing formation of disulfide bonds between the C_H_1 and C_L_ domains (Dul and Argon, 1990; Haas, 1991). Dorrington and others demonstrated that non-covalent pairing of V_H_ and V_L_ precedes formation of interchain disulfide bonds between HC and LC, thereby playing a pivotal role in regulating Ig chain assembly (Chothia et al., 1985; Hamel et al., 1986); however, our findings challenge this notion. Instead, our results indicate that V_H_-V_L_ pairing is not required for assembly of HC and LC. Rather, the critical factor governing IgG assembly and secretion is the C_H_-C_L_ pairing within the IgG molecule.

Full-size HCs were secreted effectively, and in a completely assembled form, when expressed along with either the V_κ_-C_κ_ chain or the C_κ_-only domain; this also seems to be the case for the V_H_-deficient C_γ1-3_ chain. The C_γ1-3_ chains were secreted efficiently in their assembled form when co-expressed with LCs in either the V_κ_-C_κ_ chain or C_κ_-only domain (Figure 1). Our previous findings underscored the importance of the interaction between the C_γ1_ and C_κ_ domains for Ig secretion, as co-expressing C_γ1-3_ and C_κ_ chains in HEK293F resulted in a production yield approximately 30-fold higher than that of C_γ1-3_-only, and 5-fold higher than that of the C_κ_-only domains (Kim et al., 2019). Moreover, full-size LC (V_κ_-C_κ_ chains) was secreted efficiently in both free and assembled forms, depending on the presence of the HC. Notably, the C_κ_ domain showed limited independent secretion, but was released effectively when co-expressed with HCs in an assembled form (Figures 1, 4). This implies that the association between the C_γ1_ and C_κ_ domains may induce complete folding of the C_κ_ domain, which lacks the V_κ_ region. To the best of our knowledge, this is the first study to demonstrate this mutual facilitation between the C_γ1_ and C_κ_ domains.

With respect to secretion of the HC and LC as individual entities, we found that the presence of the V domain had a markedly different effect on secretion. In the absence of the LC, the HC was not secreted, regardless of the presence/absence of the V_H_ domain (full-size HC and C_γ1-3_ chain) (Figure 1). By contrast, in the absence of HC expression, secretion of LC (V_κ_-C_κ_) was markedly higher than that of the C_κ_ chain, which was undetectable (Figure 4). In this context, during cellular IgG expression, it is probable that an unfolded V_H_ structure within HCs is inherently preferrable to maintain the unfolded state of the C_γ1_ domain of IgG, thereby aiding BiP-mediated ER retention until the C_L_ domain associates with the C_γ1_ domain. By contrast, for LCs, a folded V_L_ domain is inherently favored to facilitate C_L_ folding, although experimental confirmation is necessary.

While V_H_ and V_L_ pairing is not mandatory for Ig secretion, it is clear that the primary sequence of the V domains, when present in an Ig molecule, influences the process. This conclusion is supported by our observation that introducing an aberrant _ψ_V_κ_ domain into the Ig molecule impeded Ig secretion completely (Figure 2). Furthermore, several studies show that the V_H_ and V_L_ domain sequences have a significant impact on Ig assembly and secretion (Wu et al., 1983; Dul and Argon, 1990; Dul et al., 1992; Rocca et al., 1993; Chen et al., 1994; Horwitz et al., 1994; Martin et al., 1996; Wiens et al., 2001; Whitcomb et al., 2003; Stoyle et al., 2017; Mieczkowski et al., 2020). Single-point mutations in V_L_ can disrupt Ig assembly and secretion completely, as exemplified by the FS62 (Phe62 to Ser) mutation in the λ1 chain (Dul and Argon, 1990), the GR15 (Gly15 to Arg) mutation in the λ2 chain (Wu et al., 1983), and the YH87 (Tyr87 to His) mutation in the κ chain, in MOPC 21 myeloma cells (Dul et al., 1992). Some point mutations in V_L_ only partially block secretion (Rocca et al., 1993). LC secretion levels vary depending on their V_L_ sequence (Horwitz et al., 1994). Studies on murine T15 IgG revealed that point mutation(s) in the V_H_-complementarity-determining region 2 (CDR2) impeded assembly with LCs and subsequent secretion (Chen et al., 1994; Wiens et al., 2001). Deletion of specific residues in T15 V_H_-CDR3 restored secretion of HCs with the T15 V_H_-CDR2 mutation (Martin et al., 1996). Mutations in the framework regions (FRs) of T15 V_L_, which could not be secreted alone, restored secretion competency and enabled secretion-defective HCs with the T15 V_H_-CDR2 mutation to be secreted as assembled IgG, illustrating the compensatory effect of LC mutations on harmful HC mutations (Whitcomb et al., 2003). Yet, the exact biochemical mechanism underlying the outcomes of V domain sequence changes remains unclear. Recently, the cognate pairing preference of V_H_ and V_L_, which is a crucial factor in achieving high yields of bispecific IgG1, could be dictated by specific residues in the CDR3 loops (Fernandez-Quintero et al., 2022). Low secretion of some IgG molecules was attributed to reduced recognition of incompletely folded V_K_ by protein disulfide isomerase (PDI), which impairs disulfide bond formation within LC, ultimately leading to proteasomal degradation (Mathias et al., 2020).

Given that a previous study suggests that differences in IgG secretion levels associated with V_H_ and V_L_ sequences, it is reasonable to assume that expression of Ig comprising C_γ1-3_ and C_κ_ domains (previously designated as IgCw-γκ of 98 kDa) (Kim et al., 2019) could serve as a reference point for secretion efficiency. Further research on production of IgG-based recombinant Abs should be undertaken to investigate whether specific V_H_ and V_L_ sequences increase or reduce IgG secretion, using IgCw-γκ as a reference.

Some reports support the idea that the folding state of the C_γ1_ domain can be influenced by its upstream domain structure. Certain humanized HCs with modified V_H_-CDR3 sequences were secreted as full-size HC dimers, often referred to as HC-only Abs. These HC dimers, whether full-length or Fd dimers, were produced by CHO cells (even in the presence of cognate LCs) instead of fully assembled Ig forms (Stoyle et al., 2017; Mieczkowski et al., 2020). This phenomenon was due to promotion of Fd-Fd dimer formation by intrinsic V_H_ sequences, leading to C_γ1_ folding and formation of intrachain disulfide bonds through the robust V_H_/C_γ1_ interface. Consequently, folded C_γ1_ avoids binding to BiP and circumvents ER retention mechanisms. In another study, chimeric HCs comprising CD40-C_γ1-3_, in which the V_H_ region of the HC was replaced by human CD40, were secreted even in the absence of LCs (Capon et al., 1989). It is plausible that a specific protein located upstream of the C_γ1_ domain (whether or not it is V_H_) induces complete folding of the C_γ1_ domain, thereby bypassing ER retention mechanisms in the absence of LCs. Here, we put forth the idea that a particular protein (in our investigation, the _ψ_V_κ_ domain) upstream of the C_κ_ domain may induce structural changes in C_κ_, followed by unfavorable interactions with C_γ1_, thereby impeding IgG secretion. This, in turn, underscores the intricate interplay of antibody domains.

We only examined IgG1-based fragments to study their assembly and secretion patterns. Abs of different isotypes are likely to exhibit distinct assembly mechanisms, with IgE being a noteworthy example. In a previous study of an IgE-based fragment, we proposed the potential involvement of alternative assembly mechanisms, and chaperones other than BiP. Co-expressing C_ε1-4_ and C_κ_ chains in HEK293F cells led to secretion of individual chains without covalent assembly (Kim et al., 2022), whereas co-expressing the C_γ1-3_ and C_κ_ chains resulted in assembly and secretion of IgCw-γκ (Kim et al., 2019). Substituting the C_ε1_ domain in the Cε_1-4_ chain with the C_γ1_ domain restored secretion of assembled IgE-like molecule, designated as IgCw-γεκ, with a size of ∼130 kDa (Kim et al., 2022). Exploring the assembly process of IgE, which remains largely uncharted, would be highly fascinating.

Recombinant Ab formats based on IgG1 have been diversified for biopharmaceutical research. Our study increases our understanding of their assembly and secretion, highlighting the role of the V domains. Our data will inform the design of alternative molecular formats, potentially incorporating non-cognate HCs and LCs, for therapeutic applications.

## Data Availability

The raw data supporting the conclusion of this article will be made available by the authors, without undue reservation.

## Abbreviations

Ig, immunoglobulin; HC, heavy chain; LC, light chain; VH, variable region of the heavy chain; VL, variable region of the light chain; Cγ, constant region of gamma chain; Cκ, constant region of light chain; ERQC, endoplasmic reticulum quality control; IP, immunoprecipitation; ELISA, enzyme-linked immunosorbent assay.
